# Prevention of SARS-CoV-2 (COVID-19) transmission in residential aged care using ultraviolet light (PETRA): a two-arm crossover randomised controlled trial protocol

**DOI:** 10.1186/s12879-021-06659-7

**Published:** 2021-09-17

**Authors:** Amanda Brass, Andrew P. Shoubridge, Maria Crotty, Lidia Morawska, Scott C. Bell, Ming Qiao, Richard J. Woodman, Craig Whitehead, Maria C. Inacio, Caroline Miller, Megan Corlis, Nicolas Larby, Levi Elms, Sarah K. Sims, Steven L. Taylor, Erin Flynn, Lito E. Papanicolas, Geraint B. Rogers

**Affiliations:** 1 The South Australian Health and Medical Research Institute (SAHMRI), Adelaide, SA Australia; 2 The Microbiome and Host Health Programme, College of Medicine and Public Health, Flinders University, Bedford Park, SA Australia; 3 College of Medicine and Public Health, Flinders University, Adelaide, SA Australia; 4 Southern Adelaide Local Health Network, SA Health, Adelaide, SA Australia; 5 International Laboratory for Air Quality and Health, Queensland University of Technology, Brisbane, QLD Australia; 6 The Prince Charles Hospital, Brisbane, QLD Australia; 7 Child Health Research Centre, Faculty of Medicine, The University of Queensland, Brisbane, QLD Australia; 8 SA Pathology, SA Health, Adelaide, SA Australia; 9 Flinders Centre for Epidemiology and Biostatistics, Flinders University, Adelaide, SA Australia; 10 Registy of Senior Australians, SAHMRI, Adelaide, SA Australia; 11 School of Public Health, University of Adelaide, Adelaide, SA Australia; 12 Australian Nursing & Midwifery Federation, Adelaide, SA Australia; 13 UniSA Allied Health & Human Performance, University of South Australia, Adelaide, SA Australia; 14 Aged Care Property Services Management, Adelaide, SA Australia; 15 National Centre for Epidemiology & Population Health, The Australian National University, Canberra, ACT Australia

**Keywords:** SARS-CoV-2, COVID-19, Germicidal ultraviolet light, Residential aged care, Health care quality, Respiratory virus infection, Transmission

## Abstract

**Background:**

SARS-CoV-2 poses a considerable threat to those living in residential aged care facilities (RACF). RACF COVID-19 outbreaks have been characterised by the rapid spread of infection and high rates of severe disease and associated mortality. Despite a growing body of evidence supporting airborne transmission of SARS-CoV-2, current infection control measures in RACF including hand hygiene, social distancing, and sterilisation of surfaces, focus on contact and droplet transmission. Germicidal ultraviolet (GUV) light has been used widely to prevent airborne pathogen transmission. Our aim is to investigate the efficacy of GUV technology in reducing the risk of SARS-CoV-2 infection in RACF.

**Methods:**

A multicentre, two-arm double-crossover, randomised controlled trial will be conducted to determine the efficacy of GUV devices to reduce respiratory viral transmission in RACF, as an adjunct to existing infection control measures. The study will be conducted in partnership with three aged care providers in metropolitan and regional South Australia. RACF will be separated into paired within-site zones, then randomised to intervention order (GUV or control). The initial 6-week period will be followed by a 2-week washout before crossover to the second 6-week period. After accounting for estimated within-zone and within-facility correlations of infection, and baseline infection rates (10 per 100 person-days), a sample size of n = 8 zones (n = 40 residents/zone) will provide 89% power to detect a 50% reduction in symptomatic infection rate. The primary outcome will be the incidence rate ratio of combined symptomatic respiratory infections for intervention versus control. Secondary outcomes include incidence rates of hospitalisation for complications associated with respiratory infection; respiratory virus detection in facility air and fomite samples; rates of laboratory confirmed respiratory illnesses and genomic characteristics.

**Discussion:**

Measures that can be deployed rapidly into RACF, that avoid the requirement for changes in resident and staff behaviour, and that are effective in reducing the risk of airborne SARS-CoV-2 transmission, would provide considerable benefit in safeguarding a highly vulnerable population. In addition, such measures might substantially reduce rates of other respiratory viruses, which contribute considerably to resident morbidity and mortality.

*Trial registration* Australian and New Zealand Clinical Trials Registry ACTRN12621000567820 (registered on 14th May, 2021).

## Background

Outbreaks of SARS-CoV-2 infections (COVID-19) in residential aged care facilities (e.g. nursing homes) have proven catastrophic [1]. Rapid transmission of SARS-CoV-2 between residents, combined with the increased likelihood of severe illness or death due to resident age, comorbidities, and frailty, have resulted in the highest overall mortality rate of any population [1].

The principal infection control measures employed in most settings, including RACF, currently focus on the transmission of SARS-CoV-2 in the form of respiratory droplets. Transmission occurs via close contact with an infectious person or via contact with a contaminated surface. Prevention measures include social distancing, the use of masks, hand hygiene, and surface sterilisation. However, there have been many instances of transmission occurring despite strict adherence to such infection control strategies [2, 3]. As a result, there is growing concern that airborne transmission in the form of bioaerosols, which can remain suspended in the air for a considerable period, can contribute to transmission [4–6].

During the COVID-19 pandemic, our understanding of modes of SARS-CoV-2 transmission has evolved [7]. An increasing body of evidence supports bioaerosol-mediated spread [4–6, 8]. While the overall contribution of this transmission route remains uncertain, an airborne component of COVID-19 transmission would be consistent with other respiratory viruses, such as SARS-CoV-1, Middle Eastern respiratory syndrome coronavirus (MERS-CoV), and influenza [9, 10].

Measures to prevent airborne transmission of viral and bacterial pathogens in other clinical and non-clinical contexts, including reduced air-recirculation, air-filtration, and the use of germicidal ultraviolet (GUV) light [11], have considerable potential to reduce COVID-19 transmission in residential aged care. Such approaches can be applied in parallel to existing infection prevention measures, and implemented in a rapid, cost-effective, and non-disruptive manner. Importantly, they are not reliant on changes in the behaviour or practices of residents or staff as, for example, required for safe use of Personal Protective Equipment (PPE).

High levels of air exchange can be prohibitively expensive due to heating and cooling costs [11]. Filtration systems require ongoing maintenance to ensure efficacy and modification of existing air conditioning systems. As such, GUV has a number of important advantages over other options in the context of residential aged care facilities (RACF). GUV is highly effective in killing viral pathogens, including a 98.2% reduction in airborne influenza [12], and a 400-fold decrease in SARS-CoV-1 infectious virus [13], and is effective in other human coronaviruses, such as alpha HCoV-229E and beta HCoV-OC43 [14].

GUV technology is commercially available in several different modalities. Upper-room GUV systems direct ultraviolet rays into the upper air zone and exposing air as it circulates through natural convection. Upper-room GUV (with effective air-mixing) has been shown to reduce airborne tuberculosis transmission at a rate equivalent to implementing a high volume of mechanical air ventilation (replacing the air volume of the room 24 times per hour) [15]. Similarly, when GUV has been deployed as “in-duct” or standalone fan-based systems, it has been shown to result in significant reductions in both viral viability and rates of symptomatic respiratory infections [16]. These GUV modalities therefore allow deployment to be tailored to the particular characteristics and layout of individual RACF including proximity to residents and staff to ensure safety.

We describe a multicentre, two-arm double crossover, randomised controlled trial of a facility-level intervention involving the use of GUV light devices to reduce rates of airborne respiratory viral transmission in residential aged care settings.

## Methods/design

The *Prevention of SARS-CoV-2 (COVID-19) transmission in residential aged care using ultraviolet light* (PETRA) study is registered in the Australian and New Zealand Clinical Trials Registry ACTRN12621000567820. A publication reporting the main study outcomes will be published in accordance with the Consolidated Standards of Reporting Trials (CONSORT) statement [17].

### Study design and setting

**Fig. 1:**
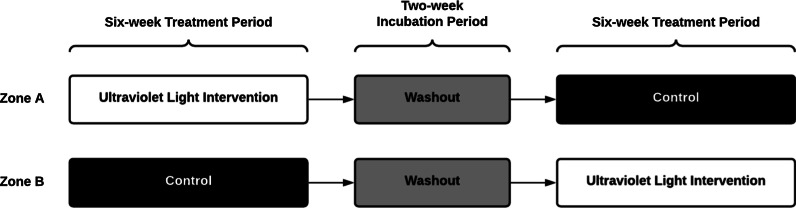
PETRA Trial Stages. Designated zones within RACF will be paired, with one zone in each pair randomised to the active or control condition for the first intervention period. Similar deployment of GUV devices in paired areas will be employed across each zone. GUV units will be switched off during control periods and run continuously during active periods. Six-week treatment periods will be divided by a 2-week “washout” period to account for viral incubation periods, before crossover to the reciprocal intervention

The PETRA study is a multicentre, two-arm double crossover, randomised, non-blinded controlled trial, with the two arms allocated to intervention order in a 1:1 ratio (Fig. 1). Building characteristics of RACF vary, including by age, layout, occupancy, rates of air change, and air ventilation systems. This variation requires the deployment of GUV devices in a manner that is facility-specific, but which also provides a consistent level of air treatment across facilities. The deployment of GUV devices focuses on communal use areas, including corridors, lobbies, dining rooms, and occasional recreation areas. No devices will be placed within resident rooms, amenities, or staff-only areas. Devices will be systematically deployed across the communal use areas of each facility to ensure consistent volumes of air are irradiated across facilities.

RACF within metropolitan and regional [18] South Australia will be considered for recruitment if they possess the ability to sub-divide communal living areas into discrete areas that enable a concurrent comparison of interventions, with the facility cohorts otherwise subject to the same facility practices (e.g. environmental cleaning, staffing, and social distancing). Each area will be randomly allocated to receive GUV light air treatment, or no air treatment, for the 6-week duration of the first period, followed by a 2-week washout period and then crossover of treatments for the second 6-week period. Cases of respiratory viral infections will be continuously monitored throughout the study, with air and environmental samples collected weekly for the duration of the study.

### Intervention

The intervention will involve the commercially available Laftech GUV appliances: UV-FLOW-C wall- and ceiling-mounted system, UV-FAN-XS wall-mounted air purifier, and UV-FAN M2/95HP air purification device (LAF Technologies, Melbourne, Australia). We will implement the combined use of two GUV approaches: wall- and ceiling-mounted GUV devices situated in locations that provide air sterilisation of shared spaces, such as dining areas, and connecting corridors and spaces between resident rooms, with additional units providing coverage to high traffic spaces, such as lift areas; and portable standalone fan-driven units used in occasional areas, such as activity halls and chapels (employed during and following area use). GUV devices will be installed in study zones within each facility. Zones will be paired within facilities, with one zone in each pair randomised to the intervention or control condition for the first period. Similar deployment of GUV devices in paired areas will be employed across each zone.

GUV units will be switched off during control periods and run continuously during intervention periods. Six-week intervention periods will be divided by a 2-week “washout” period to account for viral incubation periods, where all devices will be off, before crossover to the reciprocal intervention (Fig. 1). An open trial approach will be employed, without masking or blinding. Any changes in existing infection control practices will be recorded. The intervention will be implemented for two consecutive winters to account for variation in the prevalence of respiratory viruses.

### Outcomes

#### Primary outcome

The primary outcome will be the incidence rate ratio of combined symptomatic respiratory infections for the intervention group versus the control group. The study will utilise the existing framework for surveillance of influenza-like illness (ILI) in RACF. The guidelines published by both national (Communicable Disease Network Australia) [19] and local authorities (Communicable Diseases Control Branch of South Australia) [20] define ILI in RACF based on European guidelines [21]. The existing guidelines stipulate that residents meeting the ILI definition, including symptoms of fever, cough, or sore throat, should be discussed with the treating general practitioner and undergo testing for influenza by nucleic acid amplification using a nose and/or throat swab [19, 20]. To ensure thorough and consistent case identification, according to study definitions, the study manager/coordinator or study research nurse will liaise directly with facility staff for the duration of the study to capture this information.

During the intervention periods (Fig. 1; Table 1), identification of cases of symptomatic respiratory viral infection will be based on the existing clinical definition of ILI. RACF clinical team representatives will follow the facility’s standard surveillance program, and will complete a symptomatic respiratory infection data capture sheet, which will serve as the data collection instrument for the PETRA trial. Multiple episodes of infection in the same resident will be counted as case occasions for the same resident provided that the following criteria are met:

1. At least 2 weeks have passed since the initial case definition was met.
2. The symptoms triggering the subsequent case definition have developed acutely (within 72 h of recording).

**Table 1:** PETRA study protocol timeline

	Pre-treatment	Weeks 1–6	Weeks 7–8	Weeks 9–14	Post-treatment
RACF eligibility assessment	●				
RACF recruitment	●				
Communication with participating RACF residents, families and staff	●				
Zone allocation	●				
Design GUV device layout per zone	●				
Install GUV devices	●				
Quality control of UV-C light	●		●		
Zone randomisation	●				
Routine monitoring of respiratory viral infections		●		●	
Weekly air sampling of viral particles		●		●	
Weekly fomite sampling of viral particles		●		●	
Viral sequencing					●

*GUV* germicidal ultraviolet light; *RACF* residential aged care facilities; *UV-C* ultraviolet C

However,

3. If the same pathogen is detected for both episodes, these will be considered one episode of infection, not two.

In accordance with current guidelines, residents meeting the ILI definition should undergo subsequent pathology testing. Participating facilities will request medical officers send respiratory swab to a pathology service for diagnostic testing of viral respiratory pathogens including: *SARS-CoV-2, influenza A, influenza B, parainfluenza virus 1, 2, 3,* and *4, adenovirus, rhinovirus, human metapneumovirus,* and *respiratory syncytial virus*. Patients will have met the primary outcome based on fulfilling the symptomatic respiratory infection clinical definition, even if no swab was performed or if the swab result is negative. However, data on swab results will be obtained from pathology services. Those who meet the clinical definition and also have a positive diagnostic result will have met the definition of a confirmed symptomatic respiratory viral infection. The numbers of confirmed respiratory infection cases for each respiratory pathogen tested will be determined and analysed as a secondary outcome.

#### Secondary outcomes

*Outcome 1* Incidence rates of hospitalisation (number of hospitalisations per 100 person-days followed) for complications associated with respiratory infections. Hospital admissions, or presentations at hospital emergency departments for complications associated with acute respiratory infections, will be recorded through facility notes, including viral diagnostics and time.

*Outcome 2* Respiratory virus detection in facility air samples. In addition to assessing symptomatic respiratory viral infections, presence of viral RNA/DNA within RACF will be measured. The methodology used is not designed to distinguish between viable and non-viable viral particles, and these environmental assessments are not intended as a measure of infection risk. Rather, they provide a snapshot of the level of detectable viruses in the environment that could be missed from only testing symptomatic residents (i.e. as generated by asymptomatic staff, visitors or residents). Viral shedding by asymptomatic individuals has indeed been described in relation to both common respiratory viruses [22] and COVID-19 [23]. As an indirect measure of viral particle load, this study component will provide an additional basis to assess intervention impact.

Assessment of airborne respiratory virus presence will be performed based on air sample collection at the end of each study week (Coriolis micro liquid output air sampler; Bertin Instruments, Fortitude Valley, Queensland) [24, 25]. A sample collection protocol sufficient for the detection of aerosol-borne Respiratory Syncytial Virus (RSV) and influenza [24, 25] as well as for levels of circulating COVID-19 particles reported in healthcare environments [26] will be employed. Viral particles in the air will be determined using realtime, multiplexed versions of polymerase chain reaction assays used in the routine diagnostic respiratory assay.

*Outcome 3* Respiratory viral detection in facility fomite samples. Samples will be collected from non-absorbent fomites with pre-wetted (0.25 strength Ringer's solution) polyester-tipped swabs, using a fixed area template [27]. Presence of viral RNA/DNA in fomite samples will be determined using an identical method to that used for air samples. The selection of surfaces will include common touch-points and surfaces encountered in shared areas (e.g. lift buttons, door handles), with sample collection performed at the end of each study week for the duration of the study schedule (Table 1).

*Outcome 4* Incidence rates of laboratory-confirmed respiratory illnesses and genomic characteristics. Data will be captured on specimens sent to pathology services for respiratory virus testing during the study period. Incidence rates will be calculated for individual respiratory viruses previously described. Viral sequencing will be performed on the nucleic acid extract of laboratory-confirmed positive specimens to determine epidemiological links.

In addition to the specified study outcomes, a number of other variables will be recorded. These will include air ventilation rates, carbon dioxide levels, and facility lockdowns in response to outbreaks of notifiable infections or infections associated in rapid spread or high infectivity. For example, the identification of three or more cases of influenza-like illness occurring within 72 h in residents, or a sudden increase in influenza-like cases, or one case of influenza confirmed by any laboratory testing method in the presence of other reported influenza-like illness cases, constitutes an outbreak and triggers specific containment measures [19]. The detection of SARS-CoV-2 is referred to as a COVID-19 outbreak. Any infection-related changes in facility operation, such as facility-level vaccination rates, will also be recorded.

### Facility participation

RACF participation is at the discretion of the provider’s senior management and in consideration of the best interest of residents. Facilities will have the option to withdraw from the trial at any stage. Informed consent will not be sought from individual aged care residents. However, individual residents and their families will be communicated with prior to and throughout the trial by the study team.

### Governance

The study will comply with the National Health and Medical Research Council (NHMRC) *Australian Code for the Responsible Conduct of Research*. A Clinical Trials Memorandum of Understanding will be established between all RACF and the South Australian Health and Medical Research Institute (SAHMRI). A Study Protocol Steering Committee will be established to monitor trial progress and its ongoing ability to meet the objectives. Risk and financial management will comply with SAHMRI’s institutional policies. Consumer involvement will continue to be gathered through existing consumer reference groups at SAHMRI.

### Sample size calculation

Based on a cluster randomised two-arm double**-**crossover design, in which each RACF zone is assigned to both the intervention and the control condition twice (once for each condition in each of two consecutive respiratory infection seasons), a sample size of n = 8 RACF zones (across five facilities) with an average size of n = 40 occupied beds per zone, will provide 89% power to detect a 50% reduction in rate of symptomatic infections, i.e. five per 100 person-days in the intervention group versus 10 per 100 person-days in the control group. This calculation assumes an average of 35 days of follow-up for each subject for each of the four 6-week intervention periods providing 40 × 35 = 1400 person-days of follow-up per zone, a coefficient of variation for the zone event rate within each treatment of 50%, a 20% loss-to follow-up, and an intra-class correlation (ICC) for zones of ρ = 0.20 resulting in a variance inflation factor (VIF) of VIF = (1 − ρ)/4 = 0.2 i.e. 20% of the total number of zones and subjects required for a parallel group trial [28]. In summary, 8 zones with an average of 40 subjects per zone and 35 days follow-up per subject across each of the four separate treatment periods will be required for this double crossover trial.

### Statistical considerations

Primary analysis on the difference in infection incidence rates between the two periods will be assessed using mixed effects Poisson regression with fixed effects for the intervention group, intervention order, intervention period, and a period-to-intervention interaction term in order to assess for a possible intervention-to-period interaction effect. The logarithm of the duration of exposure to each intervention (person-days) will be included in the model as an offset term. The zone and facility will be included as random intercepts with zones nested within facilities. As a sensitivity analysis, we will also assess differences in infection incidence rates using wider time-windows for each testing period in order to account for the median incubation period of respiratory infection by up to four days, depending on the specific virus [29]. Analysis will be performed using Stata version 17. A two-sided type-one error rate of alpha = 0.05 will be used to indicate statistical significance.

## Discussion

The PETRA study aims to evaluate the feasibility and effectiveness of retrofitting GUV devices into RACF to combat respiratory virus outbreaks, including COVID-19. Despite clear potential for airborne transmission to contribute to COVID-19 outbreaks in RACF [30], current strategies employed to protect the wider community, including for more than 210,000 Australians currently living in residential aged care, do not include any measures to address this specific threat. Reductions to the risk of COVID-19 viral transmission within this vulnerable population could prevent considerable loss of life. In addition to the threat of COVID-19, this study will address the burden of other respiratory pathogens, such as RSV and influenza, that are common causes of considerable morbidity in aged care settings and transmissible via bioaerosols [6, 31, 32].

Commercially available upper-room GUV is an existing and validated technology. Previously identified as a potential solution for multi- and extensive drug-resistant pathogens [33], GUV has proven highly efficacious against airborne viruses, including influenza and SARS-CoV-1 [12, 13]. Upper-room GUV systems direct GUV light into the upper air zone and treat air as it circulates through natural convection. GUV light can also be enclosed within units that draw air into a germicidal compartment.

By assessing a potentially applicable environmental infection control strategy, we believe our study addresses a critically important public health need. Moreover, results from this study could potentially advocate for the rapid and cheap translation to additional RACF and other high-risk settings to reduce the risk of respiratory illness and mortality in our most vulnerable populations.

## Data Availability

Not applicable.

## Abbreviations

CONSORT: Consolidated Standards of Reporting Trials
COVID-19: Coronavirus associated disease 2019
GUV: Germicidal ultraviolet light
ICC: Intra-class correlation
ILI: Influenza-like illness
MERS-CoV: Middle Eastern respiratory syndrome coronavirus
NHMRC: National Health and Medical Research Council
PETRA: Prevention of SARS-CoV-2 (COVID-19) transmission in residential aged care using ultraviolet light
PPE: Personal protective equipment
RACF: Residential aged care facilities
RSV: Respiratory Syncytial Virus
SAHMRI: South Australian Health and Medical Research Institute
SARS-CoV-1: Severe acute respiratory syndrome coronavirus 1
SARS-CoV-2: Severe acute respiratory syndrome coronavirus 2
VIF: Variance inflation factor
